# The first evidence of alloparental feeding in a crevice‐nesting seabird, the little auk

**DOI:** 10.1002/ece3.11188

**Published:** 2024-04-22

**Authors:** Martyna Syposz, Marion Devogel, Antoine Grissot, Dariusz Jakubas, Katarzyna Wojczulanis‐Jakubas

**Affiliations:** ^1^ Faculty of Biology, Department of Vertebrate Ecology and Zoology University of Gdańsk Gdańsk Poland; ^2^ Littoral, Environnement et Sociétés (LIENSs) UMR 7266 CNRS—La Rochelle Université La Rochelle France

**Keywords:** *Alle alle*, alloparental care, chick adoption, dovekie

## Abstract

An alloparent is an individual that cares for a young individual, but it is not its genetic parent. This behaviour is known in many species of animals, but some groups are still underreported. Here, we documented, in camera footage, the alloparental feeding of two chicks of the little auk, a crevice‐nesting seabird. This is the first evidence of this behaviour in the little auk despite similar monitoring undertaken between 2016 and 2022 and the second record for a crevice/burrow‐nesting seabird. We compared chicks that were fed by an alloparent to other chicks from the same year and explored reasons for the behaviour in the context of seabird breeding biology.

## Graphical abstract

We documented, using camera footage, the first evidence of alloparental feeding (feeding by an individual, not being a parent) in the little auk and a second record of crevice/burrow‐nesting seabird. The set‐up allowed us a detailed insight into parental care of chicks fed by alloparents and to explore reasons for the unusual phenomenon.

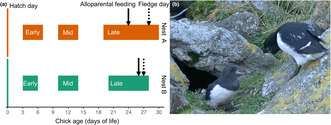

## 1 INTRODUCTION

Parental care is an energetically and time demanding process (Clutton‐Brock, 1991). To ensure the highest inclusive fitness, an adult is expected to direct its parental investment towards its offspring. However, in many animal species, not only parents but also other conspecifics take part in caring for a young (Riedman, 1982). An individual, who is not a genetic parent, providing food, protection, brooding, or other care to a conspecific is called an alloparent (Wilson, 1975). Alloparental behaviour may be adaptive, benefiting the alloparent through enhanced inclusive fitness, reciprocal altruism or parental experience. However, it can also be a costly error, often initiated by young solicitations (Riedman, 1982). Recognising the frequency and circumstances of erroneous behaviours is an important component of understanding the evolutionary drivers of alloparental behaviour. Seabirds are long‐living animals that are mainly colonial breeders, highly investing in a few offspring, making them an interesting model group in the context of alloparental care (Furness & Monaghan, 1987). Proximate factors, such as high nest density (Castillo‐Guerrero et al., 2014), high synchronicity (Eadie et al., 1988), unfavourable environmental variables (Birkhead & Nettleship, 1984) and biased sex ratio at the colony (Young et al., 2008) have been identified as amplifiers of alloparental care. Chicks of some seabirds have been reported to approach foreign nests and beg for food from parents of other chicks (Brown, 1998). Thus, aggressive behaviours of chicks or adults directed towards an unrelated chick, as well as the reduced fitness of an alloparent, should limit the appearance of alloparental behaviour (Archuby et al., 2010; Holley, 1981; Saino et al., 1994). The actual frequency and causes of alloparental behaviour in seabirds remain largely unknown. Nevertheless, alloparental care has been described in some species, either as a one‐off instance or extending to full adoption of a young (e.g. Bukaciński et al., 2000; Lecomte et al., 2006). Seabird species known to exhibit alloparental care include gulls (Brown, 1998; Bukaciński et al., 2000), terns (Cullen, 1957; Greenwell et al., 2020; Morris et al., 1991), penguins (Beaulieu et al., 2009; Jouventin et al., 1995; Lecomte et al., 2006; Wienecke, 1995), petrels (Archuby et al., 2010; Young et al., 2008), boobies (Castillo‐Guerrero et al., 2014; Guo et al., 2010), auks (common and Brünnich's guillemot, *Uria aalge* and *U. lomvia*; Birkhead & Nettleship, 1984; Gaston et al., 1995; Harris et al., 2000; Lefevre et al., 1998) and the black skimmer (*Rynchops niger*, Quinn et al., 1994). Yet, little is known about the differences between species that regularly exhibit alloparental care and those where this behaviour is rare or never documented.

In this study, we report observations (camera footage) of the feeding of two little auk (or dovekie, *Alle alle*, Alcidae family) chicks by individuals who were not their genetic parent. We compare the parental care and growth rate of these chicks with those from the same year to assess the causes of alloparental feeding, potentially induced by reduced parental care (Morris et al., 1991; Pierotti & Murphy, 1987). Finally, we discuss the reasons for the appearance of this unusual behaviour in little auks and explore it in the broader context of seabird breeding ecology.

## 2 STUDY AREA AND METHODS

### 2.1 Study area and species

The study was undertaken in a colony of little auks located in the Hornsund fjord in Spitsbergen (77°1′ N, 15°32′ E). This area is considered as the largest breeding aggregation of little auks in Svalbard (Keslinka et al., 2019). The colony density is at c.1.6 nests per 1 m^2^, with an estimated 590,000 breeding pairs of little auks (Keslinka et al., 2019; Wojczulanis‐Jakubas et al., 2008).

Little auks are long‐lived and annually return to the same partner and nest. While at the colony, they spend most of their time near the nest site area (Stempniewicz, 2001, *personal observations*). The female lays only one large egg (20% of female body mass) in a crevice and shares incubation and chick‐rearing duties with the male (Stempniewicz, 2001). During chick‐rearing, parents feed chicks within the nest, transitioning to outside feeding after c. 15 days as chicks come out to exercise wings. Fledging occurs around day 26th, often accompanied by vocalisations and male presence (Kidawa et al., 2023; Stempniewicz, 1981; Wojczulanis‐Jakubas & Jakubas, 2012).

#### 2.1.1 Video protocol

Parental behaviour of little auks was recorded between 2016 and 2022 to study the presence and behaviour of focal individuals (Grissot et al., 2019; Wojczulanis‐Jakubas et al., 2022; Kidawa et al., 2023). Cameras, placed 3–5 m from nest entrances on tripods, recorded videos in 1‐s time‐lapse mode to conserve energy and memory space. Individual colour rings on parents enabled later identification in the footage. We analysed the videos, noting the behaviour of focal individuals using previously established protocols (Grissot et al., 2019; Wojczulanis‐Jakubas et al., 2022). During each chick‐rearing period, the video recording captured a snippet of 3 days during the first (hereafter early chick rearing) and second week (mid chick rearing) of a chick's life. At the beginning of the fourth week of the chick's life (late chick rearing), the recording was resumed until the chick was no longer found in the nest for two consecutive days, i.e., considered fledged.

#### 2.1.2 Chick measurements

The nests were checked daily to establish the hatching and fledging date. We weighed a chick every 3 days using an electronic balance (OHAUS, accurate to 0.1 g). Once a chick surpassed 18 days, we deployed a metal ring. Since 2019, a long colour ring with a geolocator (GLS) attached has also been deployed. Specifically, in 2022, we deployed some chicks with a long colour ring with GLS and others without a GLS (as a part of another study investigating the effect of GLS on juveniles). Although we do not think that ringing, or GLS deployment induced alloparental behaviour, the rings of focal chicks are visible in the videos (Videos 1 and 2; one chick has a long, purple colour ring and the other a long, orange colour ring with GLS).

**VIDEO 1:** Nest A**—**an unknown adult, not identified as a parent, feeds a chick. The event is followed by a parent feeding the chick for comparison.

**VIDEO 2:** Nest B**—**an unknown adult, not identified as a parent, feeds a chick on two occasions. The events are followed by a parent feeding the chick for comparison.

#### 2.1.3 Comparing nests

In the year of the alloparental observation (2022), we video‐monitored 25 nests. Nine of them were part of an experiment, where one adult was equipped with a GPS‐logger during the chick‐rearing period. The remaining nests (*N* = 16) were treated as controls. In this group, we observed three instances of alloparental feeding in two different nests (A and B). Four nests, two with a GPS‐logger deployed parent and two without, failed to successfully rear a chick.

To investigate if reduced parental care could have elicited or contributed to allow feeding, we compared the frequency of parental feeding and chick body mass gain between the two nests with alloparental behaviour and the other control nests (nests with GPS‐logger deployed parent and failed nests were not considered). To mitigate the potential impact of yearly‐specific foraging conditions on chick rearing in little auks (Grissot et al., 2019; Jakubas et al., 2020), we exclusively utilised data from 2022.

The number of feeds was calculated for each phase of chick rearing (early, mid and late chick‐rearing) by summing all instances of parental feeding and dividing by the recording duration (i.e., resulting in a number of feeds per 24 h). We compared the frequency of feeds per 24 h between nests A, B and the 25% lower percentile of distribution of all other nests (representing reduced parental provisioning) using a bootstrap test. Additionally, we employed the *segmented* package (Muggeo, 2017) in R software (R Core Team, 2021) to identify distinct sections of chick body mass gain. This analysis revealed two sections: a steep slope associated with a chick's rapid growth phase at the beginning of chick rearing and a milder slope before fledging. We compared the two distinct slopes of chick body mass growth in 2022 to those of the chicks from nests A and B using a bootstrap test. We checked if these slopes were significantly different from the lower quartile (25%) of the distributions of slope values from the other chicks.

## 3 RESULTS

We detected instances of alloparental feeding in the little auk on three separate occasions in two nests (A and B), which were located 46 m away from each other. In both cases, unmarked adults were observed feeding the chick during the late chick‐rearing period (Videos 1 and 2). Parents were identified by colour rings and were repeatedly observed delivering food for the focal chick throughout the chick rearing period (e.g., Videos 1 and 2). We also recaptured them in the following breeding season (2023), confirming that they still had their colour rings.

From 2016 to 2022, we recorded videos in front of nests during the late chick‐rearing period for 81 nests. Additionally, we video‐recorded nests A and B for 2 years before 2022 (included in the overall number above). The same parents have been previously reported at the nest A for 7 years (since 2015). The same male from nest B was observed for 2 years (since 2020) and the female was new in 2022.

### 3.1 Description of the alloparental feeding events

The chick from nest A was observed alone, standing at the nest entrance, apparently vocalising (indicated by the repeated opening of the mouth, though no sound in the video). It was then approached by an unmarked adult with a full gular pouch, not identified as a parent. The adult temporarily left the video frame but returned and began feeding the chick outside of the nest chamber at 02:20 a.m. on 03/08/2022 (Video 1). Although the identity of the adult could not be confirmed during the two visits, given the unusual behaviour (an adult not identified as a parent approaching a chick with a full gular pouch) and the short time between the visits, the assumption that it was the same individual is the most parsimonious.

The chick from nest B was at the entrance of the nest when first approached by an unmarked adult at 00:24:48 a.m. on 12/08/2022 (Video 2). The adult had a full gular pouch and while the feeding was not visible (the nest entrance was hidden behind a rock), the adult behaved as if feeding and the size of the gular pouch visibly diminished. After 3 min, an unmarked adult, possibly the same individual (given its behaviour and overall context), approached again and fed the chick with remnants from its gular pouch. While it was impossible to observe the feeding, the focal chick (with a metal ring and a long purple colour ring) left the nest shortly after the feeding and was the only chick visible in the camera view. A similar situation at nest B repeated after 4 hours (at 04:06:09 a.m. on 12/08/2022, Video 2), where an unmarked adult with a full gular pouch approached the nest three times, feeding the chick twice.

**FIGURE 1:**
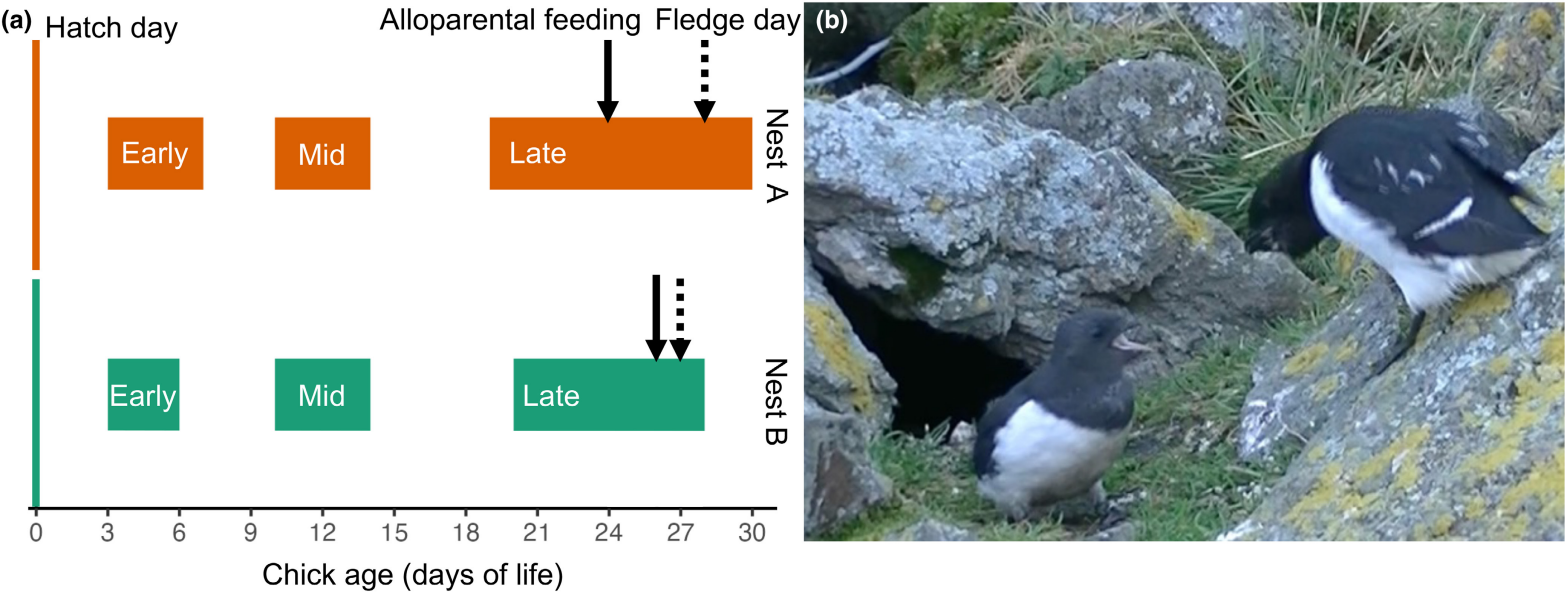
(a) Timeline of events, indicating the periods when the nests were video‐recorded (early, mid and late chick rearing phases). Alloparental feeding occurred once during the chick's 24th day of life in nest A and twice on the 26th day of life in nest B. (b) An adult, not identified as a parent (on the right), is about to feed the focal chick from the nest A (on the left).

The three feeding events by unknown individuals in both nests occurred during the last week of chick rearing. At that time, the chicks were 24 and 26 days old in nests A and B, respectively (Figure 1). Both nests were video‐recorded at early and mid chick rearing, as well as since chicks' 20th day of life until fledging. The fledging occurred on the 28th and 27th day of life in A and B, respectively.

### 3.2 Comparing nests

**FIGURE 2:**
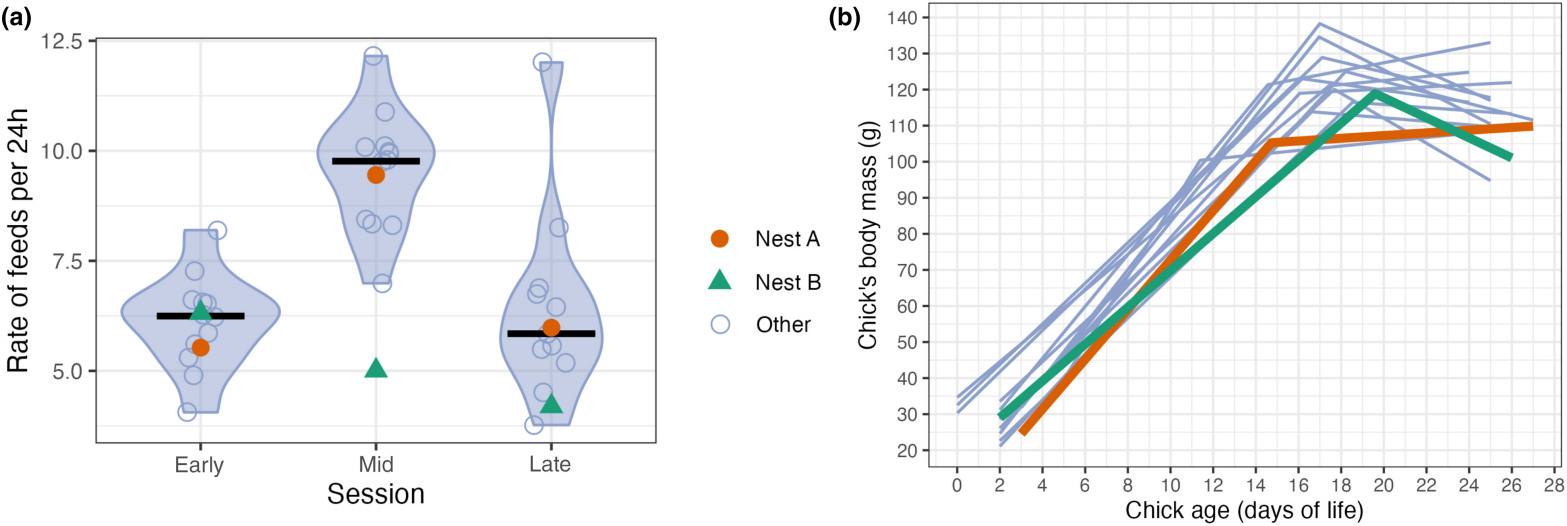
(a) Median (crossbars) and distribution (violin) of the rate of parental feedings per 24 h (data points represented by circles) separated into three phases of chick rearing (early, mid and late). The chick from nest A (orange circle) was fed as frequently as most of the chicks, fitting well within the feeding rate distribution in the colony. The chick from nest B (green triangle), was fed at a lower rate during mid chick‐rearing period compared to other chicks. (b) Estimates of slopes of body mass of all the chicks. The chick from nest A (orange line) had similar slopes of body mass growth rate as other chicks. The chick from nest B (green line) had lower slopes of growth rate than the chicks in the colony.

We tested whether the frequency of chick feeding in the nest A was significantly lower than the 25% lower percentile of the feeding rate distribution in all other nests in 2022 and we found that was not the case (Figure 2a, early: *p* = .5, mid: *p* = .79, late: *p* = .95, tested with a bootstrap procedure with 1000 iterations). The frequency of feeding the chick from nest A fit well within the feeding rate distribution of the colony. However, the feeding rate of the chick from nest B was significantly lower at the mid phase of chick rearing (mid: *p* < .001), but not at the other phases (early: *p* = .98, late: *p* = .05, a bootstrap test with 1000 iterations).

We investigated the differences in body mass between chicks fed by an alloparent and other chicks from the same year. We identified two distinct slopes in body mass gain: the first associated with a rapid increase in body mass at the beginning of chick rearing and the second representing a plateau or even decrease in the body mass before fledging. While the chick from nest A did not have significantly lower slopes than those of the last quartile (25%) of chicks from other nests in 2022 (1st slope: *p* = .59; 2nd slope: *p* = 1.00, a bootstrap test with 1000 iterations; Figure 2b), the slopes of body mass of chick from nest B were significantly lower (1st slope: *p* < .001; 2nd slope: *p* < .001, a bootstrap test with 1000 iterations).

## 4 DISCUSSION

We documented the first evidence of alloparental care in little auks, where non‐parent individuals fed two chicks on three occasions shortly before fledging. Observations were recorded via a video camera capturing parental behaviour in little auks. Similar video recordings have been ongoing since 2016 and this is the first time we noted alloparental feeding (2022). This represents only the second documented instance of alloparental care directed towards a chick in a crevice/burrow nesting seabird; the first being reported in the little penguin (*Eudyptula minor*) nesting in artificial boxes (Wienecke, 1995). Infrequent observations of alloparental care in crevice/burrow‐nesting seabirds may be attributed to the behaviour occurring below the surface or during the night when most species from this group visit colonies. Alternatively, chicks from these species might be less prone to wandering, given the high predation risk and lower likelihood of finding another nest than cliff/surface nesting birds. Furthermore, little auk adults and chicks exhibit aggression towards foreign chicks (*personal observations*), a behaviour suggested to curtail alloparental care (Brown, 1998; Spurr, 1975). Whether crevice/burrow‐nesting species are more aggressive than other seabird species remains unclear. Thus, further studies are necessary to determine if the alloparental behaviour is exhibited in these species or what might be the reasons for its absence.

We propose that the most parsimonious explanation for the observed alloparental care in this study is the specific circumstances of the feeding adults. Similar one‐off instances of alloparental care have been documented in various seabird species (Beaulieu et al., 2009; Birkhead & Nettleship, 1984; Jouventin et al., 1995; Lecomte et al., 2006). Specifically, unsuccessful breeders were reported to brood chicks, as seen in the common guillemot (Birkhead & Nettleship, 1984) or revisit the colony to feed different chicks on separate occasions, as observed in the king penguin (Lecomte et al., 2006). The adults in our study may have lost their chick due to depredation, starvation or because the chick fledged with another partner (Wojczulanis‐Jakubas et al., 2020; Wojczulanis‐Jakubas & Jakubas, 2012). A strong hormonal drive to feed a chick could lead to alloparental behaviour (Angelier et al., 2006). Furthermore, the chicks from both observed nests remained close to their nest, unlike young from other seabird species that wander between nests or roam the colony begging for food (Jouventin et al., 1995; Lecomte et al., 2006; Wienecke, 1995). This confirms that the behaviour was initially triggered by the misdirected action of the alloparent. However, it is worth noting that while the chick from nest A was fed and gained mass similarly to other chicks, the chick from nest B was fed significantly less frequently during the mid chick‐rearing period and its body mass slopes were lower compared to other chicks. Periods of reduced parental care, as observed in nest B, have previously been identified as amplifiers of alloparental care in seabirds (Morris et al., 1991; Pierotti & Murphy, 1987). Thus, we suggest that the chick's behaviour, such as loud begging (Kidawa et al., 2017), may have further attracted the already ‘confused’ adult individual.

This kind of ‘confusion’ in parents, caused by an inability to locate their chick, was previously recorded in our study set‐up. After finding a nest empty, the ‘confused’ parent spent time searching for the offspring and entered their nest multiple times. During these situations, we observed the ‘confused’ adult eating the food from their gular pouch or disappearing from the video frame and returning with a visibly smaller or empty gular pouch. While actions outside the camera view remain unknown, we have never recorded a focal parent feeding an unknown chick in those situations. Additionally, we have never observed instances where, after determining the absence of a chick in the nest, an adult returns with a full gular pouch. Hence, the possibility that the chick from the nest B was fed twice by the same individual (though uncertain due to lack of markings) is intriguing and may suggest misrecognition.

An alternative explanation for the observed alloparental care in little auks, particularly the feeding of chick from nest B on two occasions, could be that individuals mistook the focal chick for their offspring. While some colonial birds recognise the vocalisation, smell and/or behaviour of their offspring (e.g., penguins, Aubin & Jouventin, 2002), once a nest is situated in a burrow or crevice, parents rely on its location to determine which chick to feed (Becciu et al., 2021; Beecher, 1991). A recent study indicates that, despite the chick‐specific begging call of little auks, parents feed a foreign chick if it is swapped with their chick (Kidawa et al., 2023). Thus, while it is likely that the alloparental individuals could not distinguish the chick, it remains unclear why they approached the entrance of another nest. The high mobility of the chick at this stage of chick rearing and the high density of nests might have resulted in an alloparent going to a neighbouring nest and mistaking the focal chick for its own. Ultimately, it has been suggested that the cost of feeding a foreign chick might outweigh the risk of not feeding their own young (Brown, 1998; Hébert, 1988; Knudsen & Evans, 1986).

Another reason for feeding a nearby chick may be its relatedness to the alloparent. Studies have shown that some colonial seabirds group with related conspecifics (Bukaciński et al., 2000; Friesen et al., 1996, but see Brown, 1998) and thus, alloparental behaviour directed at nearby chicks could enhance the inclusive fitness of the caregiver. Little auks exhibit high site fidelity and natal philopatry (*personal observations*), potentially exacerbating the relatedness at the colony. Since we lack data on the genetics of the chicks, social parents and the unmarked individuals, we cannot exclude the possibility that observed alloparents were related to the focal chicks. Previous research indicated a low rate of extra‐pair paternity in our study site, making it unlikely that the alloparent is a biological parent of the fed chick (Wojczulanis‐Jakubas et al., 2009). However, it could be a close relative, such as an offspring of the parents from the focal nests, as only some of the chicks (3 for A and 2 for B) were ringed in recent years. Further studies, encompassing different seabird species could investigate how relatedness within clusters at the breeding colonies influences the likelihood of alloparental care in a species.

Alloparental feeding was recorded for the first time in 2022, despite detailed records on chick rearing in little auks since 2016. While long‐term monitoring is necessary for noting unusual behaviours, other factors, such as unfavourable breeding conditions, could influence a higher probability of alloparental behaviour (Guindre‐Parker & Rubenstein, 2018; Roberts & Hatch, 1994; Wienecke, 1995). Ongoing climate change negatively effects foraging behaviour, chick diet and reproduction (Descamps et al., 2022; Harding et al., 2004; Jakubas et al., 2007, 2013, 2017, 2020; Karnovsky et al., 2003; Kidawa et al., 2015; Kwasniewski et al., 2010). Although we do not observe differences in breeding success rate at our study colony (Jakubas et al., 2020, 2022), other, more subtle changes might be occurring. We will continue observations to determine if this behaviour appears more often at our study colony and we encourage other researchers to pay attention to seabirds, especially those living in burrows and crevices, for potential alloparental behaviour.

## AUTHOR CONTRIBUTIONS

**Martyna Syposz:** Conceptualization (equal); formal analysis (equal); methodology (lead); visualization (lead); writing – original draft (lead). **Marion Devogel:** Data curation (supporting); writing – original draft (supporting). **Antoine Grissot:** Data curation (supporting); writing – original draft (supporting). **Dariusz Jakubas:** Data curation (supporting); writing – original draft (supporting). **Katarzyna Wojczulanis‐Jakubas:** Conceptualization (equal); data curation (lead); formal analysis (equal); visualization (supporting); writing – original draft (supporting).

## FUNDING INFORMATION

The study was supported by grants funded by the Polish National Science Centre (SONATINA: No 2021/40/C/NZ8/00043 to MS, OPUS: No 2017/25/B/NZ8/01417 to KWJ).

## CONFLICT OF INTEREST STATEMENT

Authors declare no competing interest.

## Data Availability

The data that supports the findings of this study are available as videos of this article.
